# Hearing aid fitting and unilateral auditory deprivation: behavioral and electrophysiologic assessment

**DOI:** 10.5935/1808-8694.20120036

**Published:** 2015-10-20

**Authors:** Margarita Bernal Wieselberg, Maria Cecília Martinelli Iório

**Affiliations:** a PhD; Professor Instructor - School of Medical Sciences of Santa Casa - São Paulo); b PhD; Senior Associate Professor - Federal University of São Paulo - UNIFESP). School of Medical Sciences - Santa Casa - São Paulo

**Keywords:** event-related potentials, p300, evoked potentials, auditory, hearing aids, hearing loss, bilateral, sensory deprivation

## Abstract

The phenomenon of Late-Onset Unilateral Auditory Deprivation was first reported in 1984. However, a high number of unilateral hearing aid fittings are still carried out in cases of bilateral hearing loss, justified by non-auditory factors such as cost, vanity, misinformation and public health policies.

**Objective:**

To carry out behavioral and electrophysiological assessment of the auditory performance of adults using unilateral amplification compared with individuals exposed to bilateral symmetric auditory stimulation.

**Method:**

Thirty five adults, all with symmetric bilateral sensorineural hearing loss, regular users of unilateral hearing aid, bilateral hearing aids and not users of hearing aids, were assessed on behavioral and electrophysiological tests.

**Results:**

Variance analysis revealed that in the unilaterally fitted group, P300 latency was significantly greater in ears with auditory deprivation compared with those fitted with the hearing aid (*p* < 0.05). This same group also had poorer performance on the Sentence Recognition Test in Noise held in free field.

**Conclusion:**

These results corroborate findings in the literature showing that unilateral auditory deprivation can lead to physiological and perceptual changes.

## INTRODUCTION

The advantages of binaural hearing have been extensively communicated and documented: the possibility of functionally using both ears brings about a better understanding of speech in a noisy or reverberating environment; the capacity to locate sounds is highly dependent on the possibility of perceiving sounds with both ears at the same time^1^. Binaural hearing plays a fundamental role in monitoring and controlling numerous situations of alert and orientation in the daily routines of human beings. The interference or reduction in this skill frequently causes feelings of insecurity concerning the environment around us.

Fitting a bilateral hearing aid uses the possibility of interaction between both hearing pathways and, thus, provides the hearing impaired with the possibility of using both ears with superior quality of sound in terms of clarity, redundancy, binaural summation effect, stereophonics, no head shadow effects and, even a greater effectiveness in tinnitus suppression^1^. Considering the aforementioned, it seems coherent to suggest that the fitting of a bilateral hearing aid should be preferred, whenever there is no contraindication for it^1^, ^2^.

Long hearing deprivation periods, either partial or complete, caused by fitting an unilateral hearing aid in individuals with bilateral hearing loss, are the basis of a phenomenon which was first described in 1984^3^ and called “Unilateral Hearing Deprivation of Late Onset”. Upon prospective assessment of adults with bilateral sensorineural hearing loss, who made long use of unilateral hearing aid, noticed a significant reduction on the speech recognition of the ear which did not receive stimulation by means of a hearing aid, while on the fitted ear the indices were kept proportionally stable, causing a significant interaural discrepancy^3^, ^4^, ^5^.

The relevance of these findings became more evident since the holding of the “*1^st^ Eriksholm Workshop*” in Auditory Acclimatization and Deprivation^6^, based on which there has been guidelines, consensus recommendations and future studies published on the topic. At the time, it became clear that the detection of the possible deleterious effects of unilateral hearing deprivation would depend not only on the sensitivity of the instrument used in its assessment, but also on the systematic incorporation of its monitoring in the audiological clinical routine. With this goal, back in 1992, Gatehouse^7^ had suggested that the effect of auditory deprivation could be detected in a more precise and earlier way should more sensitive instruments had been used to recognize speech in silence. Since then, it has been recommended to include behavioral and electrophysiological tests in the assessment of the central and peripheral hearing for this end^8^.

Adults with hearing loss frequently complain of the difficulty in understanding speech in noisy environments. In order to obtain a more realistic estimate of how much communication and speech understanding are compromised, it is necessary to use assessment procedures which focus on the individual's skills to process the auditory information in situations which more closely mimic the ones he has in his daily routines. Since it is not possible to infer such difficulty based on speech tests carried out in silent environments, it is paramount to use these speech tests in the presence of noise in order to properly assess these skills^9^.

The importance of utilizing electrophysiological tests, especially the long latency auditory evoked potentials in the investigation of the auditory system, rests not only on the possibility of confirming the behavioral findings, but they also help understand the underlying physiological mechanisms and, therefore, prove to be excellent monitors of functional change^8^, ^10^. On a tutorial paper about the reorganization of the auditory system in face of amplification, Munro^8^ gathered an important unilateral amplification, on the horizons of an experimental design, which would enable a comparison of intra-subject results. By means of behavioral, electrophysiological and electroacoustic tests, such studies proved the asymmetry of response between the ear with the hearing aid and the one with auditory deprivation, suggesting that the adult auditory system may suffer perceptual and physiological changes concerning the use of a hearing aid.

Therefore, there is increasingly more evidence as to its use as an objective instrument, rather than an invasive one in functional diagnosis, in the monitoring of changes to the central auditory nervous system, in the investigation of the auditory function plasticity and in the assessment of the neuroelectric activity of the auditory pathways - from the auditory nerve all the way to the cerebral cortex^10^, ^11^. Moreover, the changes in these potentials from the situations of auditory deprivation/stimulation would reflect functional variations on the auditory pathway, bringing along possible behavioral changes, even in the absence of clear changes in subjective tests^10^.

In assessing the cortical audiological potential, the P300 component has been used with the advantage of being regularly registered in individuals with hearing loss as long as it does not prevent the patient from perceiving the rare and frequent stimuli with the same ease^12^. The drawback of using this component is on the large variability between subjects. To prevent such variability from masking the true results, some authors suggest that the individual should be his-her own control^13^.

Deprivation or asymmetry upon hearing stimulation seem to be responsible for generating a modification on the topographical representation of the auditory pathway corresponding to the auditory cortex^14^. If such assumption is true, one acceptable hypothesis from the present paper is that it should be possible to register, in cases of unilateral auditory deprivation, in a control situation (as if in a intrasubject/interaural comparison), response differences between the ear being stimulated with sounds and the one which suffered auditory deprivation.

The present paper aimed at comparing the auditory performance of individuals with bilateral hearing loss acquired in adult life, broken down into unilateral hearing aid users, bilateral users and individuals not using hearing aids by means of a behavioral assessment and the P300.

## METHOD

This study is a clinical and prospective investigation which has been approved by the Ethics in Research Committee, under protocol # 0973/07.

The present study was developed in a public service of hearing health. The series was made randomly made up by a total of 35 individuals who came to our institution for hearing assessment, for the fitting of a hearing aid or follow up of those who already used hearing aids who required a reassessment of their hearing and or concerning their performance with the hearing aid.

In order to enable the comparisons intended in this study, we created three groups of patients: one study group called “Unilateral Fitted Group” (UFG) made up of 15 participants users of hearing aids and two comparison groups called “Bilateral Fitted Group” (BFG) including 10 individuals using hearing aid in both ears and “Not Fitted Group (NFG), including 10 participants who had never before used a hearing aid. Thus, we had 35 participants in this study, adults and elderly (24 females and 11 males) with ages ranging between 48 and 90 years.

To include patients in the study, we used the following criteria: a) adult individuals older than 18 years, of both genders; b) having sensorineural hearing loss (air-bone conduction gap ≤ 10 dB in any frequency) bilateral, symmetrical, acquired at adult age; c) mean value of tonal thresholds (500, 1000 and 2000 Hz) ≤70 dB HL. For the symmetry criterion, we used the one adopted by Silman et al.^3^ in which the interaural difference in any frequency or in the speech threshold was ≤ 15 dB HL and, in the words recognition index test, the difference was ≤ 20% between ears d) no stated or overt mental, cognitive, neurological or otological disorders. Considering the patients users of hearing aids (uni or bilateral), they should use it regularly, for at least 6 hours daily and for a minimum period of 12 consecutive months.

Tympanometry was done to all the participants who had normal tympanometric pressures (+50 to -100 daPa) besides a report of no past of middle ear problems, ruling out temporary or permanent damage to the middle ear, which could impact the results.

The battery of tests employed in the three groups was based on a behavioral evaluation (tonal audiometry, speech recognition threshold in silence for monosyllable words and speech recognition in noise test) and electrophysiological assessment (P300 long latency auditory evoked potential), carried out without the patients wearing the hearing aid. Thus, the analysis did not include data on the type or brand of the hearing aid, adjusts or algorithms utilized, results from *in situ* measures or thresholds with hearing aids.

The hearing behavioral assessments were carried out in a sound proof booth, according to the ANSI 3.1 standard, from 1991. We used the GSI-61 two-channel audiometer, from Grason-Stadler, with supra-aural TDH-50P phones, calibrate according to ANSI 3.6 standards from 1989 and IEC-1988 and speakers. When the test required the use of digital playing, we used the portable NS-P4113 CD player coupled to the audiometer.

The threshold tonal audiometry was carried out by air conduction in the frequencies of 250 to 8000 Hz, with supra-aural phones and, by bone conduction in the frequencies of 500 to 4000 Hz, by means of a bone vibrator.

The Speech Recognition Index (SRI) test was carried out by means of monosyllable words which level of presentation was of 40 dB SL (sound level), based on the mean value of thresholds in the frequencies of 500, 1000 and 2000 Hz. Should such level of presentation cause discomfort, we chose to use the level of greatest comfort reported by the patient (*most comfortable level* - MCL). The material utilized for this study involved four lists with 25 monosyllable words in each, developed by Pen and Mangabeira-Albernaz,^15^ stored in a CD.

The List of Phrases in Portuguese (LPP) test^16^ was utilized in order to obtain the Speech Recognition Threshold in Noise (SRTN) and the respective Signal/Noise ratio (S/N), and it is stored in a CD. The test has a list of 25 phrases in Brazilian Portuguese, and seven other lists with ten different and balanced phrases in each. The test also has, in another channel, a noise formed by spectrums of speech, enabling both the presentation as well as the variation of the levels in which the phrases of the test are presented and the noise in an independent way. The test was used with all the participants, without the use of hearing aids, always following the same protocol, namely the SRTN was obtained from the identification of 50% of the phrases presented with a fixed and continuous noise, at an intensity of 80 dB SPL (Sound Pressure Level). In order to obtain a Signal/Noise ratio (S/N) in which the participant was able to recognize around 50% of the stimuli presented, we subtracted the SPL calculated from the SRNT from the level of noise presented, so as to obtain the S/N ratio. Thus, it was established that the S/N ratio is the difference, in dB, between the mean value of the phrase presentation level and the competitive noise. First, the assessment was carried out in a free field, with phrases and noise executed by the same speaker, at 1 meter away from the individual, at zero degree azimuth. Following, the same test protocol was employed by means of phones, with phrases and noise being presented to the same side. Thus, it was possible to assess the right and left ear (or fitted ear and ear with hearing deprivation, according to the group) separately. Different lists of phrases were utilized, one for each test situation, in order to eliminate the possibility of better performance associated to memorizing the phrases.

The electrophysiological assessment was carried out without the use of hearing aids, by means of the Long Latency Auditory Evoked Potential (LLAEP) - P300, recorded with the four channel *Biologic Systems Corp device, version 5.7*, and the auditory stimuli were presented by means of ER-3A insertion phones. The electrode position followed the International Electrode System (IES) standard 10-20^17^, namely, the ground electrode (Fpz) on the forehead, the active electrode on the cranial vertex midline (Cz), and the reference electrodes on the ear lobes (A1 = left ear and A2 = right ear). The electrical impedance of the electrodes was always below 5 Kohms and the difference between the electrodes was, at most, 2 Kohms. The exam was carried out in an sound-treated room in dim light, in a semi-dark room, with the participant comfortably seated on a reclining chair and instructed to keep still, relaxed, and awake. A number of alternate *Tone Burst* (50 ms in duration, 10 ms linear rise/fall, 30 ms plateau) stimuli were generated, at a presentation rate of 1.1/sec. The level of presentation for the stimuli varied between 70 and 85 dB HL, according to the hearing and comfort thresholds for pure tones without hearing aids. It was also required to compare and judge the balance of the intensity of the stimuli detected in both ears. In order to trigger the P300 wave, we used the rare-frequency paradigm of two tones of different frequencies (1.000 and 500 Hz, respectively) presented in a random fashion, with a likelihood of appearance between 20% and 80%, respectively. There was a total of 300 stimuli, presented to both ears, and only one record of the ipsilateral trace was captured, which was not replicated in orders to avoid interference by tiredness. The analysis of the P300, of its latency and amplitude, was carried out by three experienced examiners, independently and without them knowing the aim of the study.

The results from all the tests were recorded and plotted on a table. A descriptive and statistical analysis was carried out involving all the variables considered in the study.

This case-control study is characterized by an interaural/intra-subject comparison of the same group: the Unilateral Fitted Group, in which the hearing-aid fitted ear is compared to the one without the hearing aid, a situation in which, in the same individual, the ear stimulated with a hearing aid acts as a control for the ear deprived of hearing stimulus, which one can define as a situation of combined control. The same procedure is employed in the Bilateral Fitted Group and in the Not Fitted Group: the right ear performance is compared to that of the left ear, with the goal of looking for differences in the interaural performance of the individuals in the SRI, Recognition of Phrases in Noise and P300 tests.

Besides comparing the interaural performance of the individuals in each one of the groups, there was also a comparison of the performance among the three groups (NFG *versus* BFG *versus* NFG) in the phrases recognition in noise test employed in free field.

The inferential statistical analysis was carried out by means of the Variance Analysis test, with repeated measures or *t*-paired test. The Tukey method was employed in order to locate differences, whenever necessary. A significant level of 0.05 (5%) was employed in each hypothesis test.

## RESULTS

The ages of the participants in this study varied between 48 and 90 years (M = 70.2 years), the three tone threshold mean value for 0.5, 1 and 2 kHz was 31.6 to 69.1 dB HL (M = 50 dB); the time of hearing loss diagnosis was between three and 20 years (M = 8.3 years). In the groups of patients using sound amplification, the time interval between the initial fitting test and the current reassessment (“time using the hearing aid”) varied between two and 15 years (M = 5.8 years); the time reported of daily use of the hearing aid (“hours of daily use”) was between eight and 18 hours/day (M = 12 hours/day). The variance analysis revealed significant differences (*p* = 0.029) only concerning the time of hearing loss diagnosis between the not fitted group and those with a hearing aid (uni or bilateral).

Tables 1, 2, 3 and 4 show the results from the descriptive analysis (mean, standard deviation, maximum and minimum values) and the interaural comparison of each group using the *t Student* test. We noticed that both in the Bilateral Fitted Group (BFG) as in the Not Fitted Group (NFG), there were no statistically significant difference in the performance average when the right and left ears were compared in the SRI tests (Table 1), Phrases in Noise Test S/N (phones) (Table 2), nor in the latency variables (Table 3) and P300 LLAEP (Table 4).

**Table 1:** Mean of SRI and standard deviation (SD) for each ear and for the differences (in percentage), for the three groups. Minimum (Min) and Maximum (Max) values are available.

Not fitted (NFG)	right	10	78.8	8.4	60	88
	left	10	80.4	12.1	52	96
	difference	10	-1.6	9.7	-12	16
	right	10	68.8	8.8	52	84
Bilateral(BFG)	left	10	67.6	9.1	56	84
	difference	10	1.2	7.8	-12	16
	With aid	15	70.1	8.8	56	88
Unilateral (UFG)	In deprivation	15	66.9	14.4	40	88
	difference	15	3.2	9.1	-12	16

Right ear × Left ear (NFG e BFG) *p* = 0.920;

Ears with aid × ears in deprivation (UFG) *p* = 0.195.

**Table 2:** Mean value of the S/N ratio (phones) and standard deviation (SD) in the LSP test for each ear and for the differences (in dB), for the three groups. Minimum (Min) and Maximum (Max) values are available.

Group	Ear	n	Mean S/N	SD	Min S/N	Max S/N
Not fitted(NFG)	right	10	-1.0	4.1	-6.3	+7.7
	left	10	+0.8	4.4	-8.1	+7.0
	difference	10	-1.8	3	-7.5	+ 1.8
Bilateral(BFG)	right	10	+4.3	2.2	-0.5	+7.7
	left	10	+4.1	2.1	+0.9	+7.5
	difference	10	+0.2	3	-5.3	+4.6
Unilateral(UFG)	With aid	15	+4.8	4.2	-2.7	+ 14.6
	In deprivation	15	+4.8	3.7	-1.4	+11.6
	difference	15	0.0	3.8	-5.6	+8.6

Right ear × left ear (NFG and BFG) *p* = 0.244;

Ear with the aid × ear in deprivation (UFG) *p* = 0.979.

**Table 3:** Mean values for latency (ms), standard deviation (SD) of the P300 component for each ear and for the differences, for the three groups. Minimum (Min) and Maximum (Max) values available.

Variable	Group	Ear	n	Mean	SD	Min	Max
	Not fitted (NFG)	right	10	331.5	40.4	270.2	403.2
		left	10	332.3	40.9	268.2	390.2
		difference	10	-0.8	9.8	-23	13
	Bilateral(BFG)	right	9	342.1	41.8	283.2	424.2
Latency (ms)		left	9	338.5	44.4	278.2	422.2
		difference	9	2.6	19.2	-20	44
		With aid	14	349.3	54.2	265.2	454.2
	Unilateral(UFG)	In deprivation	14	355.0	57.0	268.2	463.2
							
		difference	14	-5.7	6.6	-18.0	5.0

Right ear × left ear (NFG and BFG) *p* = 0.797;

Right ear × ear in deprivation (UFG) *p* = 0.007.

**Table 4:** Mean values from amplitude (µv), standard deviation (SD) of the P300 component for each ear and for the differences in the three groups. Minimum (Min) and Maximum (Max) values available.

Variable	Group	Ear	n	Mean	SD	Min	Max
	Not Fitted (NFG)	right	10	5.0	3.1	0.35	10.93
		left	10	5.3	2.8	0.96	9.11
		difference	10	-0.3	1.3	-3.08	1.82
	Bilateral	right	9	4.3	2.8	0.12	10.02
Amplitude	(BFG)	left	9	4.6	2.5	0.4	9.08
(uv)		difference	9	-0.3	0.9	-1.96	0.94
		With aid	14	4.9	2.7	0.36	10.3
	Unilateral(UFG)	In deprivation	14	4.4	3.0	0.85	9.3
		difference	14	0.5	1.6	-2.0	4.4

Right ear × left ear (NFG and BFG) *p* = 0.272;

Ear with aid × ear in deprivation (UFG) *p* = 0.274.

In the analysis of the Unilateral Fitted Group (UFG) there were no differences in the mean performance between the ear in deprivation and the one fitted with the hearing aid in the List of Monosyllable Words tests (Table 1), in the Phrases in Noise Test S/R (phones) (Table 2) or on the P300 wave amplitude variable (Table 4). Inversely, the variance analysis indicated that the P300 latency was significantly higher (*p* = 0.007) in the ear with hearing deprivation, when compared to the ear with hearing aid in the UFG (Table 3).

**Figure 1:**
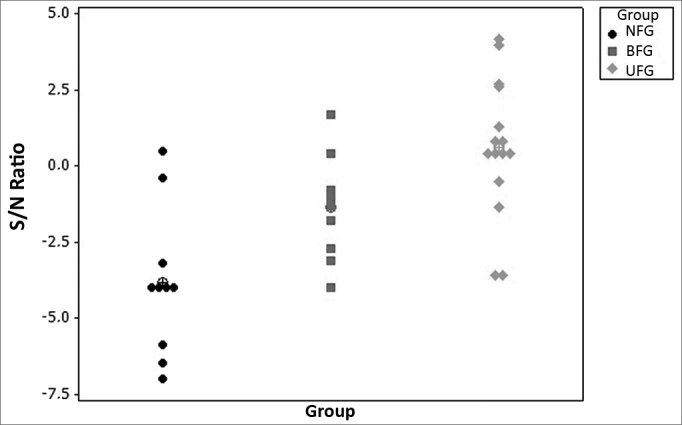
Individual and mean values of the S/N ratio (in dB) in the LSP test and that of Recognition of Phrases in Noise (free field) for the three groups.

Table 5 shows the results from comparing the performance of the three groups in the Speech in Noise test, carried out in a free field. The statistical analysis shows that the mean value of the S/N ratio in the three groups are all the same (p < 0.001) and that the NFG performance mean value was worse than in the UFG (p = 0.001) and the BFG (p = 0.086). The graphical representation on Figure 1 shows this difference with the mean and individual values in the comparison between the three groups.

**Table 5:** Mean values from the S/N ratio (free field) and standard deviation (SD) in the LSP test for the three groups (in dB). Minimum (Min) and Maximum (Max) values available.

Group	n	Mean S/N	SD	Min S/N	Max S/N
Not fitted (NFG)	10	-3.9	2.4	-7.0	+0.5
Bilateral (BFG)	10	-1.4	1.7	-4.0	+1.7
Unilateral (UFG)	15	+0.6	2.3	-3.6	+4.2

NFG × BFG × UFG *p* < 0.001;

UFG × NFG *p* = 0.001;

UFG × BFG *p* = 0.086.

## DISCUSSION

This study involved 35 individuals, and although the lack of statistical power of some measures can be associated with the small sample, they do point out important clinical arguments.

The lack of significant differences between the ear in deprivation and the ear fitted with the hearing aid in the UFG was not expected and came in disagreement with the literature^3^, ^4^, ^5^, which describes that the asymmetrical stimulation or unilateral hearing deprivation may cause a significant reduction in the SRI on the ear with hearing deprivation. This lack of difference between the ears in this group raises two pertaining questions: first, concerning the use, in clinical practice, the lists with 25 monosyllable words instead of the original lists with 50 items. The clinical use of reduced lists, with the aim of testing-retesting, brings about a statistical limitation - considering that the smaller the number of words utilized, the greater is the difference needed to exceed reliability thresholds^18^. In second, despite its unchallenged clinical value, the use of monosyllable words as the sole instrument to assess or detect a possible change in status or deterioration in speech recognition in hearing assessment may be limited and compromise the expected results.

Analyzing the results from the LPP test of phrases recognition in noise, carried out with phones, did not show differences in interaural performance in any of the three groups. The lack of statistical differences in performance between the ear in deprivation and the one with the aid in the UFG was not expected. Nonetheless, when the analysis was carried out in a qualitative and individual fashion, there as a great variability in the performance pattern of this group, and it was possible to notice that while a substantial number of individuals (10 in 15) had a systematically worse performance in the deprived ear when compared to the fitted one, some had an excellent individual performance, which may have improved the general mean result of the S/N ratio in this ear. Therefore, although this analysis did not prove to be statistically significant, it brought about clinically important information as to the difference in the performance between the ear which uses the aid and the one deprived of hearing in the UFG.

The use of the LLAEP P300 component has not received so much attention in the monitoring of changes to the central auditory nervous system as other fields in science such as psychology and neurology or in the use of the N1-P2 component of this same potential. This limited use may be justified because of the recording of large variability in the intersubject comparison of the amplitude and latency values. Its use in this study has been justified by the likelihood of broadening the reach of its use, on the feasibility of using it in patients with hearing loss and the situation in which each individual would be his/her own control (“intrasubject” assessment). Thus, it is possible to eliminate the variable inherent to the test when comparing subjects or the different age ranges, making it so that small differences may become very consistent from the experimental view point.

The variance analysis showed that only in the group using unilateral amplification we found significant interaural asymmetry. Although only 14 of the 15 subjects in this group were able to generate the P300 component, the latency of this component in the deprived ear proved to be statistically higher than what was registered in the ear fitted with the hearing aid (*p* = 0.007). The P300 latency is directly related to the time the individual needs to perceive, process and categorize the stimulus which is directly associated with the information processing speed^12^. One higher latency in the ear which suffered hearing deprivation suggests a slowing down in the response facing a possible reduction in the synaptic force, resulting in a worse neuronal synchronization or activation^19^. The reduction (or increase) in the latency of evoked potentials has been described as a neurophysiological correlation of neural plasticity and it may, often times, precede the behavioral change, which requires longer time to happen, since it may assume the integration of such changes in a perception, besides involving more central cognitive processes^11^.

This finding is in agreement with the literature, which suggests the possibility of recording changes to the morphology, latency and amplitude of the cortical auditory evoked potentials caused by stimulating (or depriving) the auditory system^7^, ^11^, ^13^.

Although the relationship between observing perceptual changes and physiological changes is largely accepted, it is still unclear as to how and how much this may happen. The latency change in the auditory evoked potentials has been described as a neurophysiological correlation of neuronal plasticity, often times preceding a behavioral change which requires longer time to show^20^. The contribution of findings by means of objective procedures, such as electrophysiological, not only confirm and reinforce the behavioral findings, but it also helps in the understanding of physiological and underlying perceptual mechanisms^7^.

The phrases recognition test in noise, carried out in a free field, was employed with the goal of assessing the use these individuals make of binaural hearing, and, as it happened in the other tests, it was carried out without the use of the hearing aids, in such a way that it would not be a bias factor depending on the degree of maintenance, type or brand of the hearing aid, adjustments of the algorithms utilized. This was the only test in which we carried out a comparative analysis of the performance “between groups”, contrary to the other tests which comparison was only “intra-aural/inter-subject”, within each specific group. The worst general mean performance in the S/N ratio of the NFG compared to the UFG (*p* = 0.001) and the BFG (*p* = 0.086) shows important information which deserve to be discussed. In a more detailed analysis, the better performance of the UFG may be associated to two determining factors for a better communication behavior: degree and time of general auditory deprivation.

Such favorable difference to this group, not only associated with the UFG as well as the BFG, is justified by the fact that participants of the UFG are still in the initial process of fitting hearing aids, showing that the diagnosis may have been carried out more recently and, therefore, register a shorter time of hearing loss (*p* = 0.029) and which concurrently, although not significantly, bear a lower degree of hearing involvement (*p* = 0.216) in comparison to the other two groups. Inversely, the worst performance of the BFG vis-à -vis the UFG may have been increased by the fact that this group had a long mean period of general hearing deprivation before fitting the hearing aids. Even taking into account these important variables and characteristics from each individual, within each group we can not refrain from showing the worst general performance of the NFG and the difficulties faced by these individuals in situations involving speech in noisy environments. The statistically significant finding of a worse performance in controlled tests is a strong evidence that the effects of such deprivation is large enough to compromise the communication performance^21^.

Evidence has shown that patients with hearing impairment, under noise, usually require a more favorable S/N ratio than a person with normal hearing would^22^. Therefore, the results from the present investigation expand these findings and suggest that individuals with unilateral hearing deprivation require an even more favorable S/N ratio when compared to those individuals with hearing loss who use or not bilateral hearing aids.

Our findings coroborate previous paper which showed that the change in the bilateral auditory processing imposed by the asymmetry in hearing stimulation during unilateral fitting of amplification may cause physiological and perceptive changes to the auditory system, which in their turn compromise situations of communication^3^, ^8^.

Along the last two decades we have had a consensus among audiologists to recommend, except in cases of contraindications, the fitting of bilateral sound amplification in cases of symmetrical bilateral hearing loss^23^. Therefore, it is preferable to avoid interventions which may compromise the balance and the benefit provided by binaural hearing^24^, as to avoid the possible effects of unilateral hearing deprivation.

It is very much true that there are reports of patients whom in certain situations of adverse hearing seem to have a better speech performance when using only one hearing aid^25^. It seems reasonable to accept that the use of bilateral amplification may not be ideal for all patients in all communication situations, especially if the user is an elderly and does it in specially difficult hearing situations, such as in the presence of competitive noise. Nonetheless, the decision for recommending the unilateral hearing aid must be based on clinical evidence, so as not to discard the benefits brought about by the bilateral stimulation of hearing by possibly solvable problems - not always associated with audiological factors. This includes investing in protocols and specific clinical situations with the goal of clarifying the true nature of performance difficulties; guarantee a minimum period of experience at home, guarantee the systematic follow up so as to overcome problems at the onset of hearing aid fitting and acclimatization; guarantee satisfactory acoustic conditions for the hearing aid, invest on education and instruction for the patient concerning the advantages of binaural hearing and a possible loss in their future communication performance stemming from unilateral hearing deprivation.

## CONCLUSION

The results from this study enable us to conclude that individuals with bilateral and symmetrical sensorineural hearing loss who use unilateral hearing aid:

Did not have interaural differences in speech recognition index (SRI) in silent environments or in the recognition of phrases in noise (LPP) when employed using phones.

Have worse signal/noise ratio performance in the medium threshold in the test of phrases in noise (LPP) carried out in the free field when compared to those individuals with binaural hearing aids and those who do not use hearing aids.

Had higher P300 potential latency in the hearing deprived ear when compared to the ear with the hearing aid.
